# Hydrological characteristics of different organic materials mulches

**DOI:** 10.1038/s41598-023-28124-y

**Published:** 2023-01-14

**Authors:** Pengfei Zhang, Menglin Xiao, Zhaorui Zhang, Yanyan Dai, Geng Liu, Masateru Senge

**Affiliations:** 1 School of Geography Science, Taiyuan Normal University, Taiyuan, 030619 People’s Republic of China; 2 Institute for Carbon Neutrality, Taiyuan Normal University, Taiyuan, 030619 People’s Republic of China; 3 Union-Infrastructure Maintenance Laboratory, Gifu University, 1-1 Yanagido, Gifu, 501-1193 Japan

**Keywords:** Plant sciences, Environmental sciences, Hydrology

## Abstract

The study aims to find the properties of three organic mulch varieties and their effects on soil moisture and crop growth. Three organic mulches: newspaper, grass, and bran were selected as the research objects, and were analyzed through double-ring infiltration and water loss tests so that water permeability and water-holding capacity of the three mulching materials could be figured out. The results showed the descending order of the three mulching treatments and non-treatment by the infiltration rate of the soil: newspaper > bare ground > grass > bran. In terms of the water-holding capacity, the three organic mulches can be ranked from high to low as newspaper, grass, and bran; by the cumulative water loss as newspaper, grass, and bran; and by water-retention capacity as bran, grass, and newspaper, respectively. By conducting regression analysis, it is found that the water-holding capacity of the mulches is related to water immersion time and the amount of water absorbed and that there is a significant logarithmic relationship between the amount of water loss and water losing time. The fitting results of the three mulches are good. Besides, a power-function relationship exists between water absorption rate and immersion time, and between water loss rate and water loss time. The water infiltration of the soil under the newspaper mulching treatment is the best, as the newspaper can help to improve soil moisture and weaken surface runoff under flood irrigation and heavy rain. Bran possesses the strongest capacity for water retention, which is beneficial to soil moisture retention in areas where sprinkler irrigation, drip irrigation, and light to moderate rain prevail. The research results can provide a basis for improving the moisture-utilization efficiency in farmlands by using organic mulches.

## Introduction

Soil moisture content fundamental to crop growth. Both excessive and deficient soil moisture can be detrimental to crop yield^1,2^. Water infiltration is a hydrological process in which water from rain and irrigation enters the soil vertically through the surface, and is mainly affected by the physical and chemical properties of the soil, rainfall intensity, topography, and surface coverage^3,4^. As one of the effective water saving measures, mulches are widely used in agricultural industry for their capabilities of retaining water and maintaining soil moisture^2^. By hindering the exchange of water vapor between the soil and the atmosphere, the mulches effectively control water loss and keep the soil at a certain moisture level, thereby improving the water-holding capacity^5,6^. Mulches can also protect the topsoil from the direct impact of water, and prolong the time of water-soil interaction to allow more water to infiltrate into the soil^7^. Mulches can adsorb water and store moisture^8^. Effects of different organic mulching materials on the physical and chemical properties of the soil and crop growth vary due to the disparity in their water permeability and water-holding capacity^9,10^.

Mulching has been an indigenous farming technique for over 300 years^11^. In other parts of the world such as Switzerland^12^, Spain^13,14^ and Belgium^15^, the original intention of using mulches was to protect soil from erosion and reduce evaporation from the top layer of the soil, thereby retaining soil moisture^16^. Wang et al.^17^ analyzed the effect of organic mulches on soil under different rainfall intensities and slope conditions through rainfall simulation experiments, and the results showed that the soil and water conservation effects of organic mulches were better than that of bare soil. Lin and Chen^18^ studied the effects of straw mulching, polyacrylamide (PAM), and grass strips on the hydraulic properties of red soil, and the results showed that straw mulches significantly improved the moisture content, the water-holding capacity, and the structure of the soil. By exploring the effects of three mulching treatments (bare slope, grassland, and straw mulching) on the physical and chemical properties of the soil, Wang et al.^19^ concluded that mulches can increase soil moisture content and that organic mulches are the best choices for water retention. Soil moisture and the physical and chemical properties of the soil can be improved by enhancing the water permeability and water-holding capacity of the mulches^20^. Guevara-Escobar et al.^21^ analyzed the water-holding capacity and permeability of organic mulches through water immersion and simulated precipitation and found that organic mulches can absorb water and retain soil moisture. Organic mulching materials (newspaper, sawdust, bark, litter, etc.) and agricultural wastes (grass, straw, rice husk, wheat bran, etc.) applied to covering the soil possess fine properties of water retention, permeability, and decomposability. They can also maintain soil moisture and prevent soil erosion, making them popular mulching materials at home and abroad^22,23^.

In general, the experimental research results sing newspaper, bran, and grass mulching treatments on farmlands have been achieved. The results showed that the three organic mulches can effectively use rainfall, inhibit soil surface evaporation, improve soil water use efficiency and promote the development of highly efficient facilities for water-saving agriculture^24–27^. However, in agricultural cultivation, many factors such as different crops, cultivation periods, external climate, soil conditions, and growth conditions will affect the mulching effect. On the contrary, the physical properties of the mulches are not subject to the external environment. To understand the effect of organic mulches on soil water content and crops, the physical properties of organic mulches, such as water permeability, water holding capacity, and water retention showed be researched first. However, there are no basic experimental results on the physical properties (water permeability, water retention) of organic mulches, making it necessary to conduct experiments to study the hydrological characteristics of the organic mulches and clarify their physical properties that are immune to cultivation conditions. In this study, double-ring infiltration, water immersion, and water loss tests were carried out under different mulching treatments (newspaper, bran, grass) to explore their water permeability, water-holding capacity, and water-loss characteristics, so that a theoretical basis for exploring soil hydrological characteristics under mulching conditions can be provided.

## Materials and methods

### Study area

The selected study area is in Yuci District, Shanxi Province (37° 23′ N–37° 54′ N, 112° 34′ E–113° 84′ E), which is lies in the east of the Loess Plateau, the north of the Shanxi-Shaanxi Basin, the northeast of the Jinzhong Basin, and the middle of the Fenhe River, a first-class tributary of the Yellow River flowing through Shanxi Province. The Fenhe River is the mother river of the district. The study area is located in a typical semi-humid warm-tempered continental monsoon climate zone with an altitude of 800–830 m characterized by an average annual temperature of about 10 °C and average annual precipitation of 400–450 mm. According to USDA's trigonometric coordinate map of soil texture classification, the soil in the test area was determined to be silty loam soil based on the sampling of the tillage layer (0–30 cm), and the average proportions of silt, sand, and clay particles were 70%, 16.5%, and 12.5%, respectively. The experiment was carried out in September 2021 after the tomato harvest and before a new round of cultivation.

### Water infiltration experiment

Double-ring infiltration test of the mulched soil was carried out on flat land in the test area without making cracks or holes in the soil^28^. The double rings were driven vertically into the ground to a depth of 10 cm, and the bottom of the double ring must be in close contact with the soil to avoid the lateral flow of water^29^. The organic mulches were then spread into the double rings. The amount of test mulching materials was determined by referring to the planting practices of local farmers. This experiment was divided into four mulching treatment groups: bran treatment (2.835 kg·m^−2^), newspaper treatment (0.229 kg·m^−2^), grass treatment (1.532 kg·m^−2^), and non-mulching treatment. 10 cm of water was injected into the outer and the inner rings simultaneously, and subsequent injections were required to meet the benchmark of 10 cm. Then, 1000 ml of water was added to the inner ring, and the time required for water infiltration to 10 cm was recorded. After repeating the process multiple times, water infiltration in the inner ring gradually slowed down and inclined to be stabilized. When the infiltration time in three consecutive water injections was nearly the same, the infiltration experiment can soon be finished. Each set of experiments was repeated 3 times, and a total of 12 water infiltration experiments were conducted.

The formula for calculating the water infiltration rate in each treatment is given below:

1 $$ V = \frac{{10Q_{n} }}{{{\text{S}}T_{n} }} $$

*V* delineates the water infiltration rate for a certain duration (mm·min^−1^). *Q*_*n*_ refers to the cumulative amount of water injected within the *n*th measurement time (ml). *S* indicates the infiltration area of the inner ring (cm^2^). *T*_*n*_ is the time interval between each of the *n*th measurements (min).

According to the experimental results, the first 10 min were determined as the initial water infiltration stage of the mulched soil. The average infiltration rate was used as the initial one, and the average infiltration was analyzed according to the initial infiltration rate, the average infiltration rate before stabilization, and the infiltration rate after stabilization.

### Experimental studies on the water-holding capacity and water-loss characteristics of organic mulches

**Figure 1:**
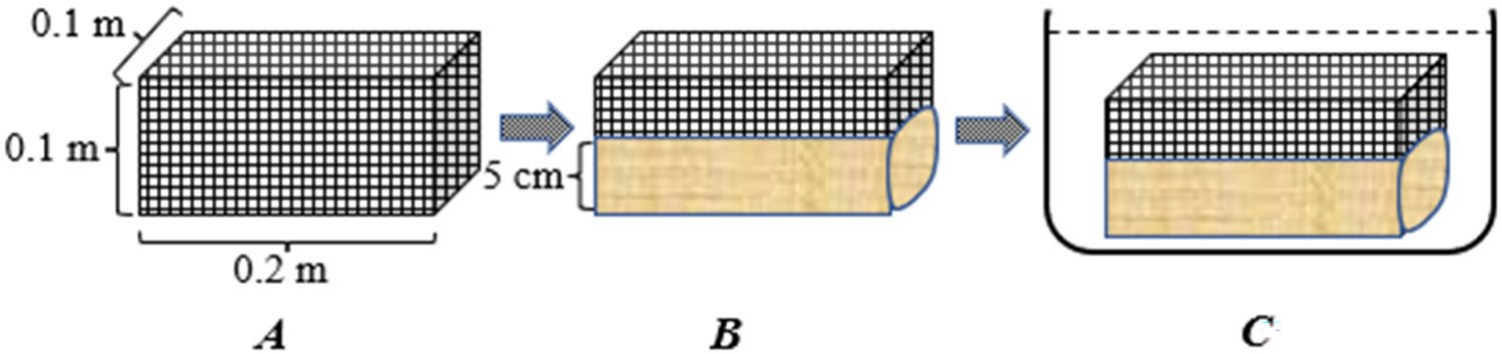
Sketch map of the water-holding capacity experiment (**A** is a special diamond mesh cage; **B** is the schematic of the 5 cm bran; **C** is the schematic of immersion of cage fitted with organic mulch).

In this paper, the method of water immersion and water loss were adopted to find out the water-holding capacity and water-loss characteristics of the mulches (Fig. 1). The naturally air-dried organic mulches were placed in a special diamond mesh cage (0.2 × 0.1 × 0.1 m) with a diameter of 0.5 mm. The amount of spread mulching material: bran (2.835 kg·m^−2^), newspaper (0.229 kg·m^−2^), and grass (1.532 kg·m^−2^), was the same as that in the water infiltration test. The thickness of the bran, newspaper, and grass were 5 cm, 0.05 cm, and 10 cm, respectively. After preparations, the covered diamond mesh cage was completely immersed in water, and was taken out after 0.5, 1, 2, 4, 8, 12, and 24 h, respectively, and was weighed immediately after being hung in the air until no more water dripped^30^. After being immersed in water for 24 h, taken out, suspended until dried (room temperature 22 °C), and weighed after 0.5, 1, 2, 4, 8, 12, and 24 h, the mulches were immediately put into a dryer. Right after drying at 65 °C for 24 h^31^. Each experiment was repeated 3 times for each group, and a total of 9 experiments were carried out. The calculation formulas are given as follows^32^:

2 $$ Q_{x} = \left( {Q_{ij} - G_{ab} } \right)/G_{ab} $$

3 $$ Vx_{{\left( {j + 1} \right)}} = \left( {Q_{j + 1} - Q_{j} } \right)/(T_{j + 1} - T_{j} ) $$

4 $$ Q_{s} = \left( {Q_{i24} - Q_{sj} } \right)/G_{ab} $$

5 $$ Vs_{{\left( {j + 1} \right)}} = \left( {Q_{j} - Q_{j + 1} } \right)/(T_{j + 1} - T_{j} ) $$

*Q*_*x*_ indicates the cumulative water-holding capacity (g·g^−1^) of the mulches over a certain immersion time and *Q*_*s*_ refers to the cumulative water loss (g·g^−1^) over a certain period of drying time. *G*_*ab*_ is the mass of the mulches in the air-dried state (g). *Q*_*ij*_ and *Q*_*i24*_ are the mass (g) of the mulches after immersion for *j* and 24 h respectively, *Q*_*sj*_ indicates the mass (g) of mulches after drying for *j* hours. *V*_*x*_ and *V*_*s*_ are the water-absorption rate and water-loss rate in a certain time (g·g^−1^·h^−1^), respectively. *T* represents the duration (h).

### Data analysis

Excel 2016 was used for data statistics and graphing in this study.

## Results

### Water infiltration rate and infiltration process under different mulching treatments

**Figure 2:**
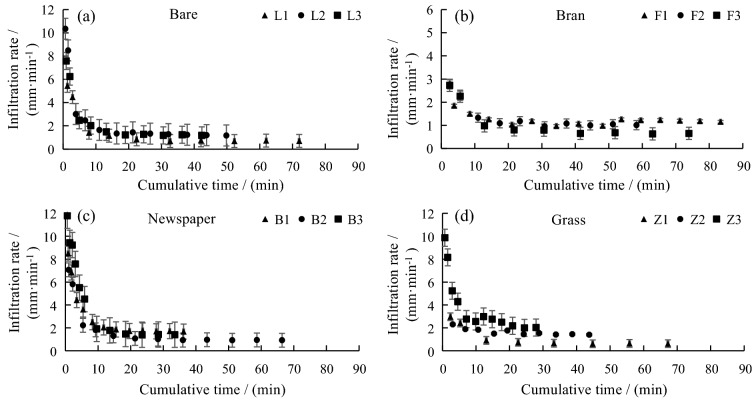
Variation characteristics of soil infiltration rate of different organic mulches.

Organic mulches act as a protective film to prevent rain or irrigation water from directly converging into the surface runoff, thus increasing water infiltration and maintaining soil moisture^33^. Soil texture is also an important factor affecting soil water infiltration. The soil texture in this study is silty loam, with silt content of 70% and sand content of 16.5%. It is characterized by low water permeability, weak water loss, and strong water-holding capacity. After being saturated by water, the silty loam can release little water by gravity, and its water-holding capacity can reach more than 90% of the water absorbed. Figure 2 shows that the infiltration rate under each treatment first increased and then decreased, and finally reached stabilization. There is a descending order of the four different mulching treatments by the effects on infiltration rate: newspaper > bare soil > grass > bran. The initial average infiltration rate of the three sampling sites on bare soil was 4.81 mm·min^−1^. The number soon decreased to 2.92 mm·min^−1^ and finally became 1.03 mm·min^−1^ after stabilization. The initial average infiltration rate of the three sites under the bran mulching treatment was 2.23 mm·min^−1^, which then fell to 1.35 mm·min^−1^ and became 0.94 mm·min^−1^ after stabilization. The initial average infiltration rate of the three sampling sites under the newspaper mulching treatment was 5.51 mm·min^−1^, which dropped to 4.02 mm·min^−1^ and fell further to the stable infiltration rate of 1.34 mm·min^−1^. Under the grass mulching treatment, the initial average infiltration rate of the three sampling sites was 3.43 mm·min^−1^. The number first dropped to 2.62 mm·min^−1^, and finally decreased to 1.37 mm·min^−1^ (Table 1).

**Table 1:** Infiltration rate characteristics of different organic mulches.

Mulches	Infiltration rate (mm·min^−1^)		
	Initial	Middle	Finally
Bare	4.81	2.92	1.03
Bran	2.23	1.35	0.94
Newspaper	5.51	4.02	1.34
Grass	3.43	2.62	1.37

### Dynamic analysis of the water-holding capacity of three varieties of organic mulches

**Figure 3:**
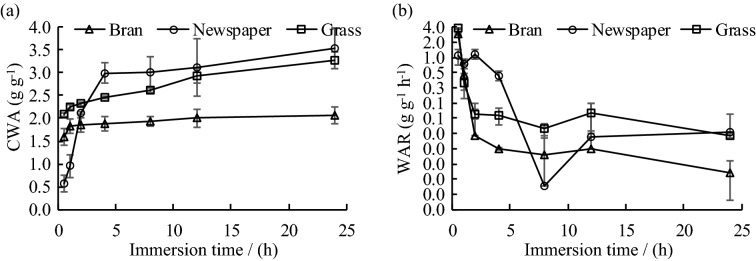
Cumulative water absorption (CWA **a**) and water absorption rate (WAR **b**) of different organic mulches.

The water-absorption capacity of organic mulches reflects their water-holding capacity. The cumulative water-holding capacity of the three organic mulches in the experiments increased with time. As represented by Fig. 3a, the water-holding capacity of bran increased rapidly in the first 1 h of immersion, and then slowed down. After 4 h of immersion, the cumulative water-holding capacity of bran mulch stabilized gradually. The water-holding capacity of newspaper mulch increased rapidly in the first 4 h of immersion and then slowed down. After 12 h, the accumulated water-holding capacity came close to stabilization. The water-holding capacity of grass material increased rapidly within the first 2 h of immersion and then became slower. After 12 h, its accumulated water-holding capacity gradually becames stable. Figure 3a also shows that after the water-holding capacity of each organic mulch tended to be stable, the cumulative water-holding capacity of newspaper material (3.52 g·g^−1^) was the best. It was 1.08 and 1.71 times the cumulative water-holding capacity of grass and bran, respectively. Figure 3b indicates that the water-absorption rate of the three organic mulches reached the maximum at the beginning, and then decreased rapidly with immersion time. The absorption rate of the newspaper began to decrease from the fourth hour onwards, while that of the bran and grass mulches decreased from the second hour onwards. After 24 h of immersion, the water-absorption rates gradually tended to 0.

Table 2 further confirms the relationship among the cumulative water-holding capacity, water-absorption rate, and water immersion time of each organic mulch on the basis of the regression analysis. The results showed that a significant logarithmic relationship exists between the cumulative water-holding capacity and the immersion time. The key equation is shown below:

$$ Q_{x} = a\ln T + b $$

**Table 2:** Regression equation for water holding and immersion time of different organic mulches.

Mulches	The relationship between Q_x_ and T		The relationship between V_x_ and T	
	Fit the relational formula	R^2^	Fit the relational formula	R^2^
Bran	Q_x_ = 0.1007In(T) + 1.75	0.888	V_x_ = 0.342 T^−1.508^	0.959
Newspaper	Q_x_ = 0.7923In(T) + 1.29	0.913	V_x_ = 0.774 T^−1.304^	0.483
Grass	Q_x_ = 0.2881In(T) + 2.18	0.913	V_x_ = 0.498 T^−1.037^	0.839

*Q*_*x*_ is the water-holding capacity of the organic mulches after a certain immersion time (g·g^−1^). *T* refers to the immersion time (h). *a* and *b* are respectively the coefficient and the constant term of the equation.

There is a significant power function relationship between the water-absorption rate and the immersion time that can be mathematically represented below:

$$ V_{x} = cT^{d} $$

*V*_*x*_ is the water-absorption rate of the organic mulches (g g^−1^·h^−1^). *T* represents the immersion time (h). *c* and *d* are respectively the regression coefficient and the index of the equation.

### Dynamic analysis of water-loss characteristics of different organic mulches

**Figure 4:**
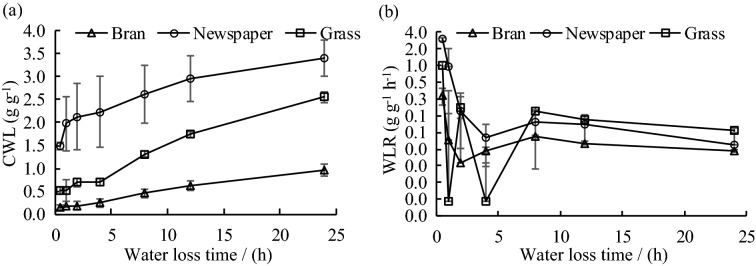
Cumulative water loss (CWL **a**) and water loss rate (WLR **b**) of different organic mulches.

The water-loss characteristic of the organic mulches also reflects the water-holding capacity of the mulches. The cumulative water loss of the three mulching materials increased with time. After 12 h of drying, the increase of water loss became slower, and after 24 h, the process continued slowly as shown in Fig. 4a. In the water loss experiment, the accumulated water loss of newspaper (3.40 g·g^−1^) was the highest. It was 1.33 and 3.54 times the amount of water loss of grass and bran, respectively. The final water holding capacity of bran (1.10 g·g^−1^) was the highest among the three kinds of mulches in the water loss experiments, and it was 1.53 and 9.17 times that of grass and newspaper. It showed that among the three organic mulches of the same weight, the water-holding capacity of bran mulching ranked first, followed by grass and newspaper. Figure 4b reveals that the water-loss rates of the three mulches were high within the first 1 h, and decreased rapidly with time until after 2 h when the water loss rates slowed down significantly. After drying for 24 h, the rates gradually approached 0.

Table 3 shows the relationship among the cumulative water loss, water-loss rate, and water-loss time of each organic mulch based on regression analysis. The results showed that there is a significant logarithmic relationship between the cumulative water loss and water-loss time. The formula of the relationship is given below:

$$ Q_{s} = e\ln T + f $$

**Table 3:** Regression equations of water loss and water loss time for different organic mulches.

Mulches	The relationship between Q_s_ and T		The relationship between V_s_ and T	
	Fit the relational formula	R^2^	Fit the relational formula	R^2^
Bran	Q_s_ = 0.1972In(T) + 0.1387	0.811	V_s_ = 0.0718 T^−0.347^	0.535
Newspaper	Q_s_ = 0.4507In(T) + 1.8092	0.954	V_s_ = 0.7188 T^−1.049^	0.956
Grass	Q_s_ = 0.5004In(T) + 0.494	0.813	V_s_ = 0.0683 T^−0.107^	0.391

*Q*_*s*_ represents the accumulated water loss of the organic mulches after they were immersed in water for a certain length of time *T* (g·g^−1^). *T* refers to the water loss time of the organic mulches (h). *e* and *f* are respectively the coefficient and the constant term in this equation.

There is a significant power function relationship between the water-loss rate and the water loss time. The formula is shown below:

$$ V_{s} = gT^{h} $$

*V*_*s*_ indicates the water-loss rates of the organic mulches (g·g^−1^·h^−1^). *T* is the immersion time (h). *g* and *h* are the regression coefficient and the index of the equation respectively.

## Discussion

Organic mulches can reduce surface evaporation, improve soil moisture as well as water-utilization efficiency, and allow for the reuse of wastes^34^. High water permeability indicates that rainwater and irrigation water can quickly infiltrate and be stored in the soil^35^. A high-water infiltration rate implies that water can be maximally used in the soil. Besides, erosion from ground runoff can be reduced, and soil moisture content can be increased^36^. The water-holding capacity of organic mulches depends on the amount or thickness of mulches on the soil^37^. Organic mulching materials can differ in quality and quantity, as well as their water permeability, water-absorption capacity, water-holding capacity, and water-loss characteristics. Owing to their water retention ability, water permeability, and decomposability, mulching materials can affect the soil moisture environment. In addition, some organic mulches can retain water from rainfall and irrigation as sponges, thus preventing runoff and providing water at the time of crop requirement^20^.

### Characteristics of water infiltration of the soil under different organic mulching treatments

The infiltration rates of the soil under various mulching treatments were high in the beginning and decreased gradually with time until they reached stabilization. It was decided by the effects of mulching and the water content of the soil. In the early stage, the mulching materials and the soil were in a dry state and thus caused a high water-infiltration rate. As the water content of organic mulches and soil increased with time, the water-infiltration rate lowered. Finally, when the water content of mulches and the soil was saturated, the infiltration rate went stabilized. The water permeability of the mulching materials is mainly affected by the thickness, composition, water-holding capacity, and degree of decomposition^38,39^. León et al.^37^ indicate that mulches of excessive thickness would absorb too much water to allow it to infiltrate into the soil. Soil texture is fundamental factor affecting soil water infiltration. Generally, the coarser the texture, that is, the higher the sand content, the better the permeability^40^. Some studies show that the water permeability is closely related to the silt content in the soil, which is in the range of 35–80%, and that the permeability coefficient is negatively related to the silt content^40,41^. In this study, the silt content in the soil makes up 70%, and the sand content occupies 16.5%, so the water permeability of silty loam is low. Through the water infiltration experiments, it was found that the water permeability of newspaper mulching was the best, followed by bare soil, grass, and bran. The reason for the higher water-infiltration rate of soil under the newspaper mulching treatment may due to its thinness, softness, and a strong capacity for water absorption. Generally, paper is porous and hygroscopic as it contains 70% pores. It also expands and shrinks with the change in water content^34^. On the contrary, the lower water-infiltration rates of the soil under grass and bran mulching treatments are attributed to their thickness. In the early stage of irrigation, the grass and bran mulches will absorb a lot of water and form a water-absorbing layer on the soil surface, in which bran will swell with water and thus keep water from infiltrating into the soil^42^.

### Dynamic changes in the water-holding capacity of different organic mulching materials

The water-holding capacity of the organic mulches increased with time, while the water-absorption rate decreased with time. It was because of the low surface water potential and the high water-absorption rate of the dry mulches when they were just immersed in water. After a long time of immersion, the water potential and the water-absorption rate gradually decreased, while the water-holding capacity went nearly saturated. The results showed that the water-holding capacity of the newspaper is the best, followed by grass and bran. This indicated that organic materials vary in their water-holding capacity^43^. The water accumulation reflects the differences in the water absorption properties of different organic mulching materials^44^. The newspaper mulch possesses the best water-absorption capacity and can effectively reduce surface runoff in a short time, while bran has a less good water-absorption capacity that is not conducive to soil and water conservation. According to the experimental data on water-holding capacity and water loss of three organic mulches of newspaper, grass and bran at 24 h, a logarithmic relationship was found between the cumulative water-holding capacity and the water immersion time, and a power function relationship exists between the water-absorption rate and the water-immersion time.

### Dynamic changes in the water-loss capacity of different organic mulching materials

The accumulated water loss of different organic materials increased with time, while the water-loss rate decreased with time. Mulches released more water in the early stage of the experiment, so the water-loss rates were high. However, as the mulches lost more water, the water-loss rate decreased. The water-loss rates of the mulches reflect their water-retention capacity^45^. A large amount of water loss from newspaper mulching indicated the poor water-retention capacity, and the less water loss of bran indicates better water retention. It might be explained by the characteristics of newspaper, such as smooth surface, low porosity, low actual water retention, and low water loss. These features can cause high water evaporation after irrigation^46^. Bran has a strong capacity for water retention and slow water loss characteristic due to its inherently fine porous structure. Under bran mulching treatment, irrigation water will not only reach the soil surface, but will be retained in the bran layer. As the soil water infiltrates the soil and is absorbed by the crops, the water in the bran will be slowly released into the soil to maintain the soil moisture. The thickness of the bran mulch of about 5 cm and its soft and smaller particles that had poor porosity hinders the water vapor exchange between the atmosphere and soil surface. When irrigation water fills the pores between bran particles (high water retention) after irrigation, it will almost cut off the circulation channel between soil and atmosphere, thus reducing the surface soil moisture evaporation loss. In addition, crusting of the bran layer surface, further seriously affected water vapor circulation between the soil surface and atmosphere. According to the experimental data on water loss of three organic mulches of newspaper, grass, and bran at 24 h, a logarithmic relationship was found between the cumulative water loss and the water-loss time, and a power function relationship was found to exist between the water-loss rate and the water-loss time^47,48^.

A conclusion was drawn by comparing the water-holding capacity and the water-loss characteristics of different organic mulching materials. Although the water-holding capacity of newspaper mulch is better, its water-loss is more than that of the other two types of mulches, which leads to poor water retention. Contrastingly, though the water-holding capacity and water-loss characteristics of grass and bran mulches are proved to be worse than that of newspaper mulch, their water-retention capacity is better. The water-retention capacity of bran is the best, followed by grass. Therefore, when the newspaper is used for mulching, water will permeate through the newspaper. As the water-retention capacity of the newspaper is relatively weak, the infiltration rate of the soil under the newspaper mulching treatment is high. Meanwhile, the bran mulch, which performed well in water retention, can lead to a lower water-infiltration rate of the soil. The results of water infiltration experiments are consistent with that of the experiments on water-holding capacity and water-loss rates of the three mulches.

## Conclusion

The results showed the descending order of the three mulches treatments and non-treatment in terms of the infiltration rate: newspaper, bare soil, grass, and bran treatments. The cumulative water-holding capacity of the three organic mulches increased with time. It is ranked from high to low as newspaper, grass, and bran. For water loss, the three mulches are ranked as newspaper > grass > bran.

In conclusion, the results of the water infiltration test of the soil are consistent with that of the indoor experiments on the water-holding capacity and water-loss characteristics of the three organic mulches. This proved that the water permeability of the newspaper is better than that of the others. Therefore, using newspaper mulch under flood irrigation or heavy rain conditions can help to reduce surface runoff and increase soil moisture. Also, the fine water retention of bran is conducive to improving soil moisture in areas where sprinkler irrigation, drip irrigation, and light rain prevail.

In this study, quantitative research was conducted to understand the hydrological characteristics of silty sandy loam mulched with organic materials under cultivation conditions, which is helpful to choose appropriate mulching materials for farmlands. The next step will be to study the effect of organic mulching threshold on soil hydrology.

## Data Availability

All data generated or analyzed during this study are included in this published article.

## References

[1] Chukalla A, Krol M, Hoekstra A (2015). Green and blue water footprint reduction in irrigated agriculture: Effect of irrigation techniques, irrigation strategies and mulching. Hydrol. Earth Syst. Sci..

[2] Zhu G (2021). Effects of plastic mulch on soil water migration in arid oasis farmland: Evidence of stable isotopes. CATENA.

[3] Lucas-Borja M (2018). Short-term changes in infiltration between straw mulched and non-mulched soils after wildfire in Mediterranean forest ecosystems. Ecol. Eng..

[4] Ezenne G, Obalum S, Tanner J (2019). Physical-hydraulic properties of tropical sandy-loam soil in response to rice-husk dust and cattle dung amendments and surface mulching. Hydrol. Sci. J..

[5] Chen N, Li X, Shi H, Yan J, Hu Q, Zhang Y (2021). Assessment and modeling of maize evapotranspiration and yield with plastic and biodegradable film mulch. Agric. For. Meteorol..

[6] Farzi R, Gholami M, Baninasab B (2017). Water-retention additives’ effects on plant water status and some physiological parameters of two olive cultivars under reduced irrigation regimes. Acta Physiol. Plant.

[7] Khan M (2016). Effect of slope, rainfall intensity and mulch on erosion and infiltration under simulated rain on purple soil of south-western Sichuan Province China. Water.

[8] Parhizkar M (2021). Effects of length and application rate of rice straw mulch on surface runoff and soil loss under laboratory simulated rainfall. Int. J. Sediment. Res..

[9] Cheng H (2020). Effects of different mulching and fertilization on phosphorus transformation in upland farmland. J. Environ. Manag..

[10] Zheng J, Fan J, Zou Y, Chau H, Zhang F (2020). Ridge-furrow plastic mulching with a suitable planting density enhances rainwater productivity, grain yield and economic benefit of rainfed maize. J. Arid Land.

[11] Li X (2003). Gravel–sand mulch for soil and water conservation in the semiarid loess region of northwest China. CATENA.

[12] Nachtergaele J, Poesen J, van Wesemael B (1998). Gravel mulching in vineyards of Southern Switzerland. Soil Tillage Res..

[13] Martínez-Zavala L, Jordán A (2008). Effect of rock fragment cover on interrill soil erosion from bare soils in Western Andalusia Spain. Soil Use Manag..

[14] Govers G, Van Oost K, Poesen J (2006). Responses of a semi-arid landscape to human disturbance: A simulation study of the interaction between rock fragment cover, soil erosion and land use change. Geoderma.

[15] Poesen J, De Luna E, Franca A, Nachtergaele J, Govers G (1999). Concentrated flow erosion rates as affected by rock fragment cover and initial soil moisture content. CATENA.

[16] Qiu Y, Xie Z, Wang Y, Malhi S, Ren J (2014). Long-term effects of gravel—Sand mulch on soil organic carbon and nitrogen in the Loess Plateau of Northwestern China. J. Arid Land.

[17] Wang B (2021). Efficient organic mulch thickness for soil and water conservation in urban areas. Sci. Rep..

[18] Lin L, Chen J (2014). The effect of conservation practices in sloped croplands on soil hydraulic properties and root-zone moisture dynamics. Hydrol. Process..

[19] Wang H, Gao J, Li X, Wang H, Zhang Y (2014). Effects of soil and water conservation measures on groundwater levels and recharge. Water.

[20] Iqbal R (2020). Potential agricultural and environmental benefits of mulches—A review. Bull. Natl. Res. Cent..

[21] Guevara-Escobar A, Gonzalez-Sosa E, Ramos-Salinas M, Hernandez-Delgado G (2007). Experimental analysis of drainage and water storage of litter layers. Hydrol. Earth Syst. Sci..

[22] Ranjan P, Patle G, Prem M, Solanke K (2017). Organic mulching—A water saving technique to increase the production of fruits and vegetables. Curr. Agric. Res..

[23] Luna L, Vignozzi N, Miralles I, Solé-Benet A (2017). Organic amendments and mulches modify soil porosity and infiltration in semiarid mine soils. Land Degrad. Dev..

[24] Suo G, Xie Y, Zhang Y, Luo H (2019). Long-term effects of different surface mulching techniques on soil water and fruit yield in an apple orchard on the Loess Plateau of China. Sci. Hortic..

[25] Kader M, Senge M, Mojid M, Ito K (2017). Recent advances in mulching materials and methods for modifying soil environment. Soil Tillage Res..

[26] Zribi W, Aragüés R, Medina E, Faci J (2015). Efficiency of inorganic and organic mulching materials for soil evaporation control. Soil Tillage Res..

[27] Chakraborty D (2008). Effect of mulching on soil and plant water status, and the growth and yield of wheat (*Triticum aestivum* L.) in a semi-arid environment. Agric. Water Manag..

[28] Lai J, Ren L (2007). Assessing the size dependency of measured hydraulic conductivity using double-ring infiltrometers and numerical simulation. Soil Sci. Soc. Am. J..

[29] Elaoud A, Hassen H, Salah N, Masmoudi A, Chehaibi S (2017). Modeling of soil penetration resistance using multiple linear regression (MLR). Arab. J. Geosci..

[30] Zhou Q (2018). Comparing the water-holding characteristics of broadleaved, coniferous, and mixed forest litter layers in a Karst Region. Mt. Res. Dev..

[31] Carnol M, Bazgir M (2013). Nutrient return to the forest floor through litter and throughfall under 7 forest species after conversion from Norway spruce. For. Ecol. Manag..

[32] Li Y (2015). Differential water and soil conservation capacity and associated processes in four forest ecosystems in Dianchi watershed, Yunnan Province China. J. Soil Water Conserv..

[33] Luna L, Miralles I, Lázaro R, Contreras S, Solé-Benet A (2017). Effect of soil properties and hydrologic characteristics on plants in a restored calcareous quarry under a transitional arid to semiarid climate. Ecohydrology.

[34] Kader M, Nakamura K, Senge M, Mojid M, Kawashima S (2019). Soil hydro-thermal regimes and water use efficiency of rain-fed soybean (Glycine max) as affected by organic mulches. Agric. Water Manag..

[35] García-Moreno J, Gordillo-Rivero Á, Zavala L, Jordán A, Pereira P (2013). Mulch application in fruit orchards increases the persistence of soil water repellency during a 15-years period. Soil Tillage Res..

[36] Chang B, Wherley B, Aitkenhead-Peterson J, McInnes K (2021). Effects of urban residential landscape composition on surface runoff generation. Sci. Total Environ..

[37] León J, Echeverría M, Badía D, Martí C, Álvarez J (2013). Effectiveness of wood chips cover at reducing erosion in two contrasted burnt soils. Zeitschrift Für Geomorphologie, Supplementary Issues.

[38] Cerdà A (2018). Policies can help to apply successful strategies to control soil and water losses. The case of chipped pruned branches (CPB) in Mediterranean citrus plantations. Land Use Policy.

[39] Girona-García A (2021). Effectiveness of post-fire soil erosion mitigation treatments: A systematic review and meta-analysis. Earth Sci. Rev..

[40] Medinski T, Mills A, Fey M (2009). Infiltrability in soils from south-western Africa: Effects of texture, electrical conductivity and exchangeable sodium percentage. S. Afr. J. Plant Soil.

[41] Mirbabaei S, Shahrestani M, Zolfaghari A, Abkenar K (2013). Relationship between soil water repellency and some of soil properties in northern Iran. CATENA.

[42] Liao Y, Cao H, Xue W, Liu X (2021). Effects of the combination of mulching and deficit irrigation on the soil water and heat, growth and productivity of apples. Agric. Water Manag..

[43] Xie J, Su D (2020). Water-holding characteristics of litter in meadow steppes with different years of fencing in inner Mongolia China. Water.

[44] Zagyvai-Kiss K, Kalicz P, Szilágyi J, Gribovszki Z (2019). On the specific water holding capacity of litter for three forest ecosystems in the eastern foothills of the Alps. Agric. For. Meteorol..

[45] Dunlop M, Blackall P, Stuetz R (2015). Water addition, evaporation and water holding capacity of poultry litter. Sci. Total Environ..

[46] Haapala T, Palonen P, Korpela A, Ahokas J (2014). Feasibility of paper mulches in crop production: A review. J. Sci. Food Agr..

[47] Kim J, Onda Y, Kim M, Yang D (2014). Plot-scale study of surface runoff on well-covered forest floors under different canopy species. Quat. Int..

[48] Kahlon M, Lal R, Ann-Varughese M (2013). Twenty-two years of tillage and mulching impacts on soil physical characteristics and carbon sequestration in central Ohio. Soil Tillage Res..

